# Efficacy and safety of VNS therapy or continued medication management for treatment of adults with drug-resistant epilepsy: systematic review and meta-analysis

**DOI:** 10.1007/s00415-022-10967-6

**Published:** 2022-01-16

**Authors:** Sarah Batson, Rohit Shankar, Joan Conry, Jane Boggs, Rodney Radtke, Stephen Mitchell, Francesca Barion, Joanna Murphy, Vanessa Danielson

**Affiliations:** 1 Sarah Batson, Mtech Access Limited, 30 Murdock Road, Bicester, OX26 4PP Oxfordshire England; 2 Neuropsychiatry, Peninsula School of Medicine, University of Plymouth, Plymouth, England; 3 Children’s National, Washington, District of Columbia USA; 4 Wake Forest Baptist Health, Winston-Salem, NC USA; 5 Duke University School of Medicine, Durham, NC USA; 6 Pricing, Health Economics, Market Access and Reimbursement (PHEMAR), LivaNova, London, England; 7 Global VP, PHEMAR, LivaNova, London, England

**Keywords:** Anti-seizure medication, VNS therapy, Meta-analysis, Drug-resistant epilepsy, Seizure frequency

## Abstract

Vagus nerve stimulation (VNS) Therapy® is an adjunctive neurostimulation treatment for people with drug-resistant epilepsy (DRE) who are unwilling to undergo resective surgery, have had unsuccessful surgery or are unsuitable for surgery. A systematic review and meta-analysis were conducted to determine the treatment effects of VNS Therapy as an adjunct to anti-seizure medications (ASMs) for the management of adults with DRE. A literature search was performed in August 2020 of the Medline®, Medline® Epub Ahead of Print, Embase, and the Cochrane library databases. Outcomes examined included reduction in seizure frequency, seizure freedom, ASM load, discontinuations, and serious adverse events (SAEs). Comparators included best medical practice, ASMs, low-stimulation or sham VNS Therapy. Four RCTs and six comparative observational studies were identified for inclusion. Against comparators, individuals treated with VNS had a significantly better odds of experiencing a ≥ 50% reduction in seizure frequency (OR: 2.27 [95% CI 1.47, 3.51]; *p* = 0.0002), a ≥ 75% reduction in seizure frequency (OR: 3.56 [95% CI 1.59, 7.98]; *p* = 0.002) and a reduced risk for increased ASM load (risk ratio: 0.36 [95% CI 0.21, 0.62]; *p* = 0.0002). There was no difference in the odds of discontinuation or the rate of SAEs between VNS versus comparators. This meta-analysis demonstrated the benefits of VNS Therapy in people with DRE, which included improvement in seizure frequency without an increase in the rate of SAEs or discontinuations, thereby supporting the consideration of VNS Therapy for people who are not responding to ASMs and those unsuitable or unwilling to undergo surgery.

**Supplementary Information:**

The online version contains supplementary material available at 10.1007/s00415-022-10967-6.

## Introduction

Epilepsy is a common neurological condition, affecting approximately 50 million people globally [1]. At least 30% exhibit drug-resistant epilepsy (DRE) and continue to suffer seizures despite treatment [2]. DRE is defined by the International League Against Epilepsy (ILAE) as failure of adequate trials of two tolerated, appropriately chosen and used anti-seizure medication (ASM) schedules (whether as monotherapies or in combination) to achieve sustained seizure freedom [3].

People with DRE experience significantly more comorbidities, including depression, seizure-related injuries, and neurological deficits than those without epilepsy or with epilepsy that responds to treatment [4, 5], and have significantly higher mortality risk [6, 7]. DRE is also associated with sudden unexplained death in epilepsy (SUDEP) which represents a major cause of death in this population [8–10]. People with DRE have substantially higher healthcare costs than those who are seizure-free, including costs associated with medical investigations, treatment costs, emergency room visits, hospitalisations, and outpatient care [18–20]. In addition, people with DRE frequently report feeling stigmatised because of their epilepsy [11].

It has been reported that greater than 30% of people with DRE remain uncontrolled despite the availability of new ASMs, and this statistic has not changed over the last 20 years. [12, 13]. For people who fail to experience sufficient seizure reduction with pharmacologic therapy, alternative approaches include epilepsy brain surgery [14], diet modification [15], and neurostimulation devices [16–19], including Vagus Nerve Stimulation Therapy® (VNS Therapy®) [20, 21].

While for many people with DRE brain surgery can be curative and result in seizure freedom, with up to 52% of people remaining seizure-free (apart from simple partial seizures) 5 years post-intervention [22, 23]. However, not all individuals are suitable candidates, and uptake of surgery is limited by hesitancy, in part due to fears of postoperative permanent neurological deficits [24].

VNS Therapy represents a commonly used neurostimulation option for people with DRE who do not wish to undergo cranial surgery or laser interstitial thermal ablation, who have had unsuccessful surgery or are not suitable for surgery (including individuals with intellectual impairment who may be unable to understand and consent to a non-reversible procedure) [25–27]. VNS is a minimally invasive extracranial device which delivers mild, intermittent electrical pulses to the vagus nerve which then stimulates areas in the brain responsible for seizures [28, 29]. This results in a reduction in seizure frequency [20, 21]. VNS Therapy® has been in clinical use in Europe since 1994 [30] and in the USA since 1997 [31].

This systematic literature review (SLR) and meta-analysis examined the treatment effects of VNS Therapy at up to 2 years as an adjunct to ASMs for the management of adults with DRE based on the most up-to-date evidence from randomised controlled trials (RCTs) and comparative observational studies.

## Materials and methods

### SLR

An SLR was conducted on the 25th of August 2020 (in alignment with the Preferred Reporting Items for Systematic reviews and Meta-Analyses [PRISMA] checklist) [32] to identify relevant clinical studies (RCTs and observational comparative studies) comparing VNS Therapy as an adjunct to ASMs with relevant comparator arms in adults with DRE followed by a meta-analysis to determine treatment effects for several efficacy and safety outcomes.

The SLR searched the electronic databases of Medline®, Medline® Epub Ahead of Print (In-Process & Other Non-Indexed Citations), Embase, and the Cochrane library to identify relevant clinical studies (RCTs, controlled clinical studies, and prospective registries) examining VNS Therapy and other interventions of interest for the management of patients with DRE. Additional searches of congress proceedings from the past 3 years (American Epilepsy Society [AES], Congress of Neurological Surgeons [CNS] Annual Meeting, European Congress on Epileptology [ECE], International Epilepsy Congress [IEC], International Neuromodulation Society [INS] Congress), reference lists of included publications, and Health Technology Assessment (HTA) bodies were conducted to identify relevant evidence. Search terms are listed in the Supplementary Materials. Citations were screened by a single analyst and independently checked by a second analyst; any discrepancies were resolved by consensus. Outcome data were extracted to a Microsoft® Excel spreadsheet.

For this analysis, the eligibility criteria included comparative clinical studies of VNS Therapy for the management of DRE conducted predominantly in an adult population (i.e., > 50% of individuals were aged ≥ 18 years). Eligible comparators to VNS Therapy were: (1) best medical practice (BMP), (2) continuation of stable ASM regimen, (3) addition of ASM, and (4) low-stimulation VNS Therapy (parameters defined in Table 3).

### Data collection and risk of bias assessment

General patient/participant demographics were extracted, such as age at time of implant, sex, type of seizure and baseline seizure frequency. Outcomes of interest included reduction in seizure frequency, seizure freedom, ASM load, discontinuations, and serious adverse events (SAEs).

Quality (risk of bias) assessment of RCTs was conducted using the seven-criteria checklist provided in Sect. 2.5 of the National Institute for Health and Care Excellence (NICE) single technology appraisal (STA) user guide for RCTs [33]. Observational studies were assessed using the quality assessment tool for quantitative studies of the Effective Public Health Practice Project (EPHPP) [34].

### Meta-analysis

Evidence synthesis was conducted via pairwise meta-analyses based on RCT and comparative observational studies. While observational comparative evidence is of lower quality compared with RCTs due to the inherent bias within such studies, their inclusion was deemed appropriate as observational comparative studies provide longer follow-up compared with RCTs. Pairwise meta-analyses were conducted for the outcomes of interest previously described. For one RCT (PuLsE) [35], which reported outcomes up to 2-year post-surgery, the outcome results were restricted to the 12-month timepoint. The 12-month results were included in the meta-analysis. Outcomes for this study were restricted to 12 months to facilitate data comparisons as all other RCTs included in the meta-analysis had shorter followups (range: 3.5–6 months).

### Statistical analysis

Evidence synthesis was conducted via pairwise meta-analyses based on RCT and comparative observational studies where available. The pairwise meta-analyses were conducted in RevMan 5.3. Heterogeneity was assessed using the chi-squared and I-squared statistics. Results were presented as an odds ratio (OR) or weighted mean difference with 95% confidence intervals (CIs).

## Results

### SLR

#### RCTs and comparative observational studies

**Fig. 1:**
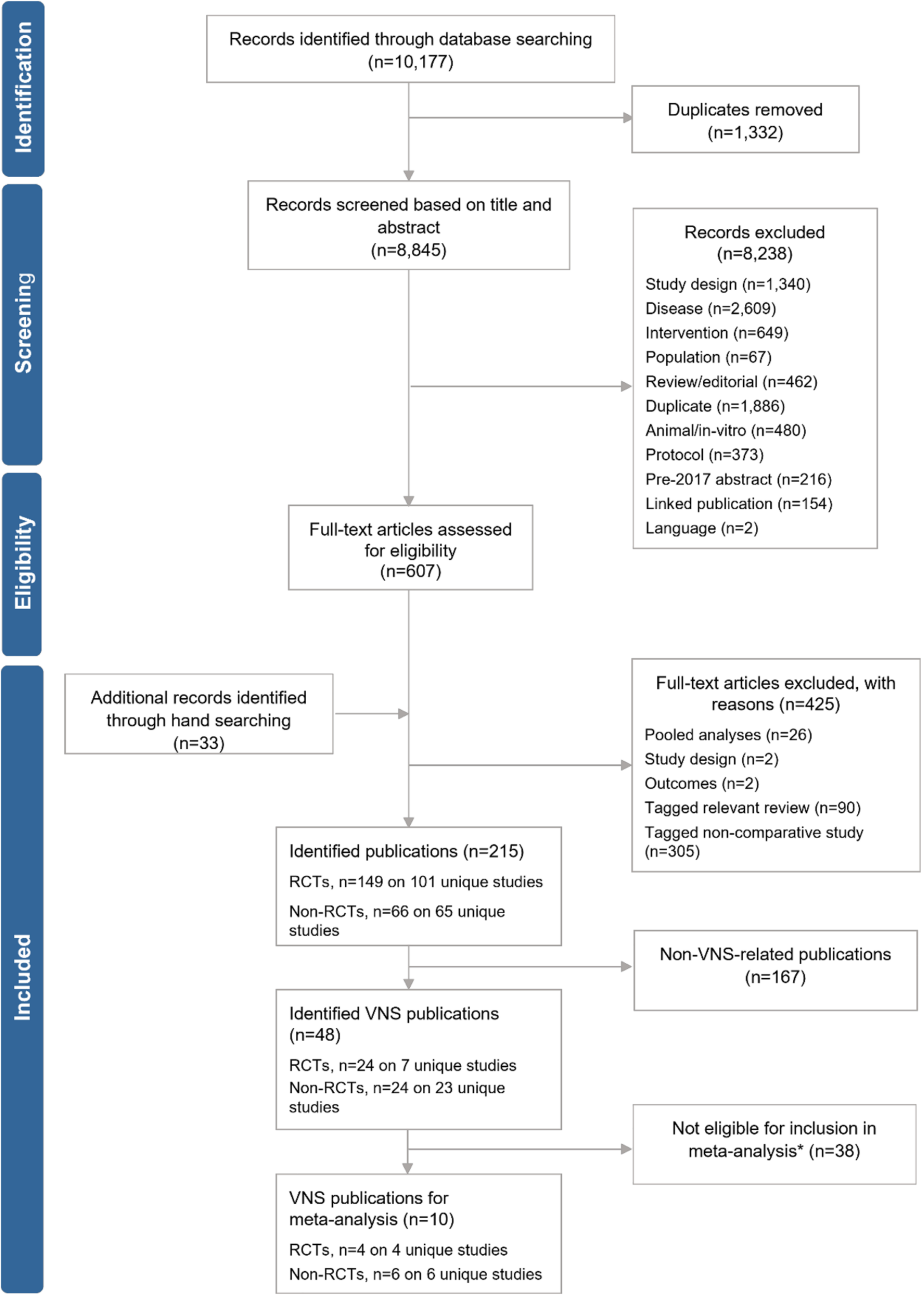
PRISMA flow diagram. Abbreviations: *RCT* randomised controlled trial, *VNS*, Vagus Nerve Stimulation. *Primarily due to publications including non-relevant comparators, no outcomes of interest or publications were superseded by a linked publication

A total of 48 publications (on 30 unique studies, see Supplementary Materials Table 1) were identified for potential inclusion. In total, 38 VNS Therapy studies did not meet eligibility criteria for inclusion in this meta-analysis (due to publications including non-relevant comparators, no outcomes of interest or publications were superseded by a linked publication); 10 studies were identified for inclusion (four RCT studies [4 unique publications] and six comparative observational studies [5 unique publications]) (Fig. 1). The publication dates ranged between 1993 and 2015.

Four primary study publications from four RCTs in an adult population were included in the analysis; 13 publications linked to these RCTs were excluded as they did not report outcomes of interest or were superseded by the primary study publication [36–47]. A single RCT study (with two linked publications; see Supplementary Materials Table 1) was excluded due to unclear reporting of the enrolled population (i.e., proportion of adults) [48, 49]. A total of 12 adult comparative observational study publications were excluded for the following reasons: no comparator (*n* = 4) [50–53], population of interest (*n* = 2) [54, 55], reported no outcomes of interest (n = 5)[56–60] and superseded by a linked primary publication (*n* = 1)[61].

### Study and participant characteristics

Study design and baseline participant characteristics of the VNS Therapy studies are shown in Tables 1 and 2. The study duration ranged from 3 to 4.5 months for the majority of RCTs [20, 21, 62], with one RCT study lasting 24 months [35]. The study duration for comparative observational studies was typically > 12 months (range: 3–32 months) [63–68]. Where reported, the mean participant age ranged 32–41 years for the RCT studies [20, 21, 35, 62] and 25–40 years for comparative observational studies. Disease duration was only reported by half of the studies included (*n* = 5), with the mean duration ranging between 20 and 23 years for RCT studies [20, 21] and 17–26 years for comparative observational studies [63, 66, 68]. Mean seizure frequency ranged from 0.6–1.7 and 0.1–3.5 seizures per day for RCT and non-comparative observational studies, respectively. The mean number of drugs used ranged between 2 and 3 for both RCT [20, 21, 35] and comparative observational studies [64–66, 68]. Three studies compared a low-stimulation setting (control arm) plus background ASMs with a high-stimulation setting; the difference between the low- and high-stimulation parameters is provided in Table 3. Rationale for using the low-stimulation included the facilitation of titration, ethical reasons, inclusion of an active control group and to permit a double-blind trial design [20, 62, 69]. The majority of studies (*n* = 7) compared VNS Therapy with a continuation of the participants’ current ASM regimen; only one comparative observational study reported the type of ASMs participants were taking [65]. None of the included studies made a specific comparison between VNS Therapy and the latest generation of ASMs (e.g., those licensed in the last two decades [i.e., lacosamide, cannabidiol, brivaracetam, perampanel etc.]). VNS is an option for people with DRE who are unsuitable for epilepsy surgery, have had unsuccessful surgery or are unwilling to undergo resective surgery. Only three out of ten of the included studies provided rationale for the use of VNS, reasons included unsuitability for surgery and patient choice [63, 64, 66].

**Table 1:** Summary of included RCTs

Author/Publication	Location	Treatment length	Duration of follow-up	Comparators	Sample size, *n*	Age at implant mean (SD [range])	Sex, F (*n* [%])	Mean duration of disease, years	Mean no. of ASMs used at baseline	Seizure types,^†^ *n* (%^‡^)	Seizure frequency	Outcomes assessed
Ryvlin [35] (PuLsE)^§^	Canada, Europe	2 years	2 years	VNS + BMP	54	38 ± 13	24 (50)	NR	3.5 (SD: ± 1.17)	Structural/metabolic: 26 (54) Unknown: 22 (46)	Median: 5 per week (range: 1 to 123)	Seizure frequency (including ≥ 50%) HRQoL ASM usage Adverse events
				BMP (ASM)	58	41 ± 11	21 (44)		3.2 (SD: ± 1.22)	Structural/metabolic: 26 (54) Unknown: 22 (46)	Median: 4 per week (range: 1 to 42)	
E-05 (Handforth [20])^§^	US	3 months	3.5–4 months	VNS high stim	95	32.1 (10.8 [13–54])	46 (48.4)	23	2.2 (SD: ± 0.7)	CPS or partial + secondarily generalised, ± other seizure types	Mean: 1.59 per day (SD ± 1.96) Median: 0.58 per day	Seizure frequency (including ≥ 50%, ≥ 75% and seizure free) ASM usage Quality of life Adverse events Discontinuations
				VNS low stim	103	34.2 (10.1 [15–60])	59 (57.3)		2.1 (SD: ± 0.7)		Mean: 0.97 per day (SD ± 0.94) Median: 0.51 per day	
E-03 (Salinsky [21]) ^§^	Europe, North America	3 months	3.5–4 months	VNS high stim	54	33.1 (NR)	21 (39)	23.1	2.09	SPS: 24 (44.4%) CPS: 50 (92.6%) Partial secondarily generalised: 38 (70.4%)	Mean 1.49 per day (SD: NR) Median: 0.73 per day	Seizure frequency (including ≥ 50%, ≥ 75% and seizure free) Adverse events
				VNS low stim	60	33.5 (NR)	22 (37)	20.0	2.08	SPS: 25 (41.6%) CPS: 58 (96.6%) Partial secondarily generalised: 33 (55.0%)	Mean 1.71 per day (SD: NR) Median: 0.82 per day	
Landy [62]	US	3–4.5 months	6.5 months	VNS high stim	5	NR	NR	NR	NR	NR	NR	Seizure frequency Adverse events
				VNS low stim	4							

Abbreviations: *ASM* anti-seizure medication, *BMP* best medical practice, *CPS* complex partial seizures, *HRQoL* health-related quality of life, *NR* not reported, *RCT* randomised controlled trial, *SD* standard deviation, *SPS* simple partial seizures, *VNS* vagus nerve stimulation

^†^May be counted in more than one type; ^‡^Where applicable; ^§^Cyberonics-sponsored VNS study (Cyberonics is owned by LivaNova)

**Table 2:** Summary of included comparative observational studies

Author /Publication	Location	Treatment length	Comparators	Duration of Follow-up	Sample size, n	Age at implant mean (SD [range])	Sex, F (*n* [%])	Mean duration of disease	Mean no. of ASMs used	Seizure types,^†^ *n* (%^‡^)	Seizure frequency	Outcomes Assessed
Gonen [64] (Comparative retrospective observational study)	Israel	≥ 1 year	VNS + ASM	5.67 years	33	> 18 years of age	14 (42.4)	NR	2.91 (SD: ± 0.95)	NR	Mean: 3.52 per day (SD 0.67)	Seizure frequency, ASM usage
			ASM	4.04 years	47		26 (55.3)		2.32 (SD: ± 0.98)		Mean: 3.15 per day (SD 0.72)	
Hoppe [66] (Comparative, Case control)^§^	Germany	> 2 years	VNS + BMP (ASM)	6.8 years (SD 2.1, range 2–13)	20	39.8 (SD: 10.2)	8 (40)	25.7 (SD: 13.4)	2.47 (SD: ± 0.77)	SPS: 8 (40%) CPS: 18 (90%) SGS: 10 (50%)	Mean: 68.4 (SD 206.3) per month	Seizure frequency, ASM use and tolerability, VNS tolerability HRQoL, ASM use, adverse events
			BMP (ASM)		20	39 (SD: 8.5)	8 (40)	21.0 (SD: 9.2)	2.24 (SD: ± 0.44)	SPS: 7 (35%) CPD: 17 (85%) SPS: 12 (60%)	Mean 8.2 (SD 10.4) per month	
Marrosu [65] (Comparative Prospective case control)	Italy	1 year	VNS implant	1 year	10	33.1 (23–44)	4 (40)	NR	1.9	CPS	Mean 156 (range: 98–212) per trimester	Seizure frequency, GRD distribution
			No implant		7	30.8 (21–42)	3 (43)		1.9		Mean 150 (range 88–206) per trimester	
Boon [63] (Comparative Prospective Cohort)	Belgium	Average 26 months [range 12–57 months]	VNS + ASM	29 months (range: 12–57)	25	31 years (range: 12–49 years)	NR	21 years (range: 2–50 years)	NR	SPS + SG: 15 (60%) CPS + SG/SPS: 3 (12%) CPS + SG/atonic: 2 (8%) CPS: 5 (20%)	Mean: 21 per month (range: 2 to 180)	Epilepsy-related direct medical costs, Seizure frequency Number and dosage of ASMs, Number of hospital admission days clinic visits and laboratory tests
			Surgery	28 months (range, 12–54)	35	32 (range: 10–60 years)				SPS + SG: 1 (3%) CPS + SG: 17 (49%) CPS: 14 (40%) GTC: 1 (3%) CPS/atonic: 1 (3%) CPS + SG/atonic: 1 (3%)	Mean: 6 per month (range: 1 to 17)	
			ASM only	25 (range: 12–48)	24	34 years (range: 5–71 years)				CPS: 7 (29%) CPS + SG: 10 (42%) SPS/CPS + SG: 1 (4%) GTC: 2 (8%) SPS/CPS: 2 (8%) CPS + SG/psych: 2 (8%)	Mean: 12 per month (range: 1 to 30)	
Tatum [68] Comparative Prospective case control)	US	13.2 months	VNS	13.2 months (NR)	21	24.8 (range: 4–51)	9 (43)	17.0 (range: 4–45)	2.81 (range: 1–5)	NR	NR	Seizure frequency, ASM dose and usage, QoL
			Control (ASM)		21	26.1 (5–57)	12 (57)	19.9 (range: 3–46)	2.38 (range: 1–4)			
Harden [67] (Comparative—Prospective cohort)^§^	US	3 months	VNS	3 months (SD 1.7)	20	39 (SD: 9.1 [range: 20–58])	14 (70)	NR	NR	CPS: 12 (60%) CP + secondarily GTC: 5 (25%) Primary GTC: 3 (15%)	Mean: 16.2 (SD 19.4) per month	Seizure frequency, Medication side effects, HRQoL
			BMP (ASM)	3.8 months (SD 1.6)	20	40.2 (SD: 13.3 [range: 24–69])	14 (70)			CPS: 10 (50%) Secondarily GTC: 5 (25%) CP + Secondarily GTC: 5 (25%)	Mean: 3.2 (SD 7.4) per month	

Abbreviations: *ASM* anti-seizure medication, *BMP* best medical practice, *CPS* complex partial seizures, *GABA*_*A*_ gamma-aminobutyric acid, *GRD* GABA_A_ receptor density, *GTC* generalized tonic–clonic seizures, *HRQoL* health-related quality of life, *NR* not reported, *psych* psychogenic nonepileptic seizures, *RCT* randomised controlled trial, *SD* standard deviation, *SG* secondary generalisation, *SPS* simple partial seizures, *SZ* seizure, *VNS* vagus nerve stimulation

^†^May be counted in more than one type; ^‡^Where applicable; ^§^Cyberonics-sponsored VNS study (Cyberonics is owned by LivaNova)

**Table 3:** Summary of high and low stimulation parameters

VNS therapy parameter	Landy 1993		E-03 (Salinsky 1995)		E-05 (Handforth 1998)	
Device setting	Low	High	Low	High	Low	High
Output current (mA)	0.5–3.0	0.5–3.0	0.25–2.75	0.25–3.0	1.2 (avg)	1.3 (avg)
Signal frequency (Hz)	1–2	20–50	1–2	20–50	1	30
Pulse width (µsec)	130	500	130	500	130	500
Signal on time (sec)	30	30–90	30	30–90	30	30
Signal off time (min)	60–180	5–10	60–180	5–10	180	5

### Participants experiencing ≥ 50% reduction in seizure frequency

**Fig. 2:**
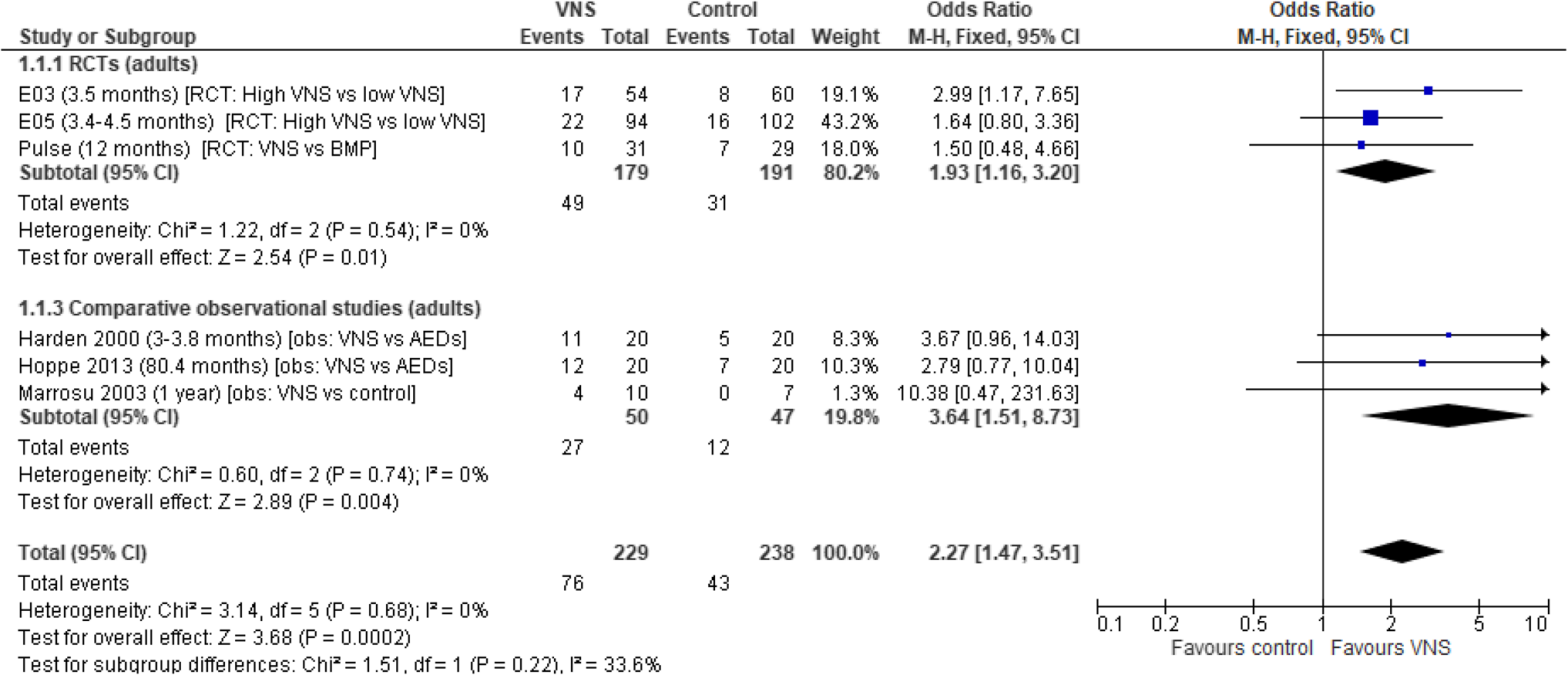
Participants experiencing ≥ 50% reduction in seizure frequency. Abbreviations: *ASM* antiepileptic drug, *BMP* best medical practice, *CI* confidence interval, *RCT* randomised controlled trial, *SD* standard deviations, *VNS* vagus nerve stimulation

A total of six studies (three RCTs and three comparative observational studies) were included in the analysis. Overall, the pooled odds ratio (based on the results of RCTs and comparative observational studies) for experiencing ≥ 50% reduction in seizure frequency was statistically significantly greater in adult participants undergoing VNS Therapy compared with low stim VNS Therapy/BMP/ASM (OR: 2.27 [95% CI 1.47, 3.51]; *p* = 0.0002). A similar statistically significant result was observed when results were pooled by study type (RCTs: OR 1.93 [95% CI 1.16, 3.20], *p* = 0.01; observational comparative studies: OR 3.64 [95% CI 1.51, 8.73], *p* = 0.004). Low levels of heterogeneity were observed between studies (Fig. 2).

### Participants experiencing ≥ 75% reduction in seizure frequency

**Fig. 3:**
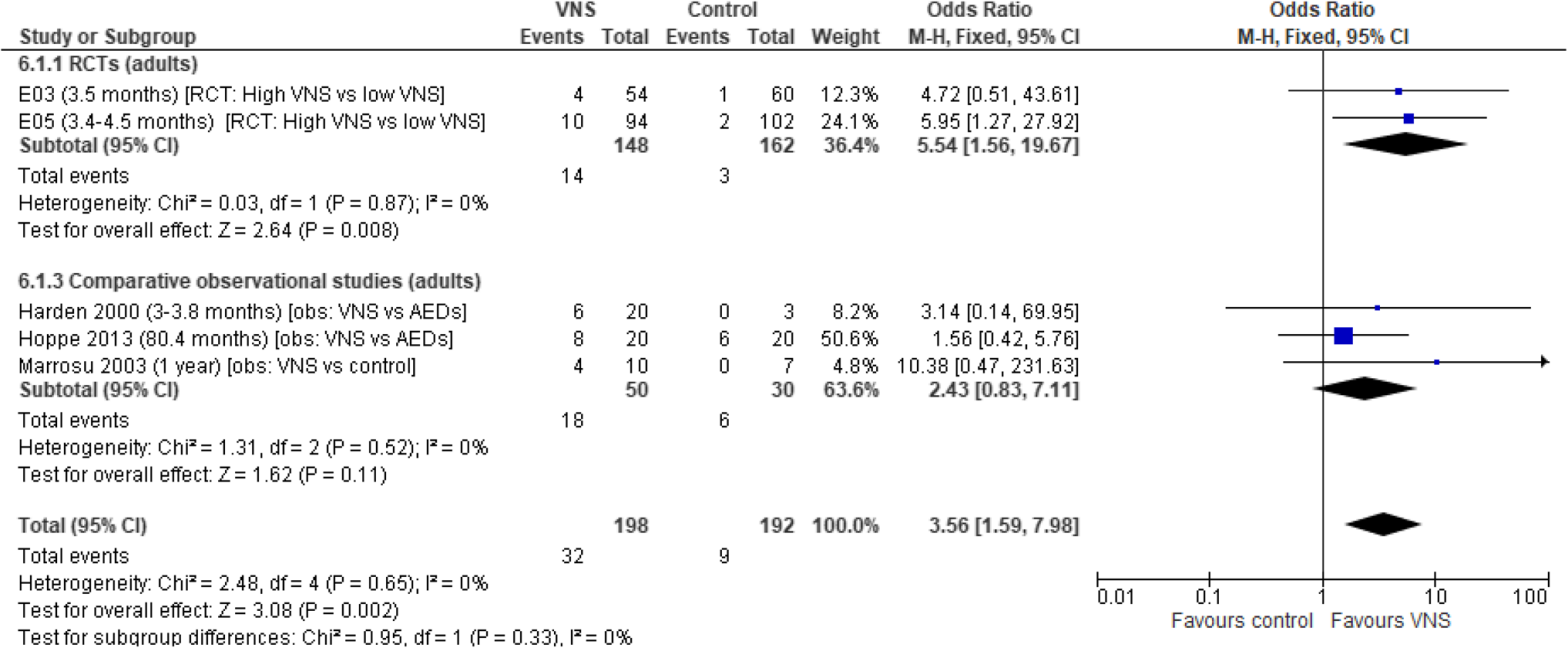
Participants experiencing ≥ 75% reduction in seizure frequency. Abbreviations: *ASM* antiepileptic drug, *CI* confidence interval, *RCT* randomised controlled trial, *SD* standard deviations, *VNS* vagus nerve stimulation

Five studies (two RCTs and three comparative observational studies) were included in the analysis. In the pooled analysis, the odds of experiencing a ≥ 75% reduction in seizure frequency were more than three times greater in adult participants undergoing VNS Therapy compared with low-stimulation VNS Therapy/ASM (OR: 3.56 [95% CI 1.59, 7.98]; *p* = 0.002). A similar statistically significant result was observed for pooled RCT studies (OR 5.54 [95% CI 1.56, 19.67]; *p* = 0.008); pooled results for comparative observational studies were not statistically significant (OR: 2.43 [95% CI 0.83, 7.11]; *p* = 0.11). A trend for a greater VNS Therapy treatment effect in RCTs at a shorter follow-up time (OR 5.54 [95% CI 1.56, 19.67]) compared with observational data at a longer follow-up time (OR: 2.43 [95% CI 0.83, 7.11]) was observed. Low levels of heterogeneity were observed between studies (Fig. 3).

### Participants that are seizure free

**Fig. 4:**
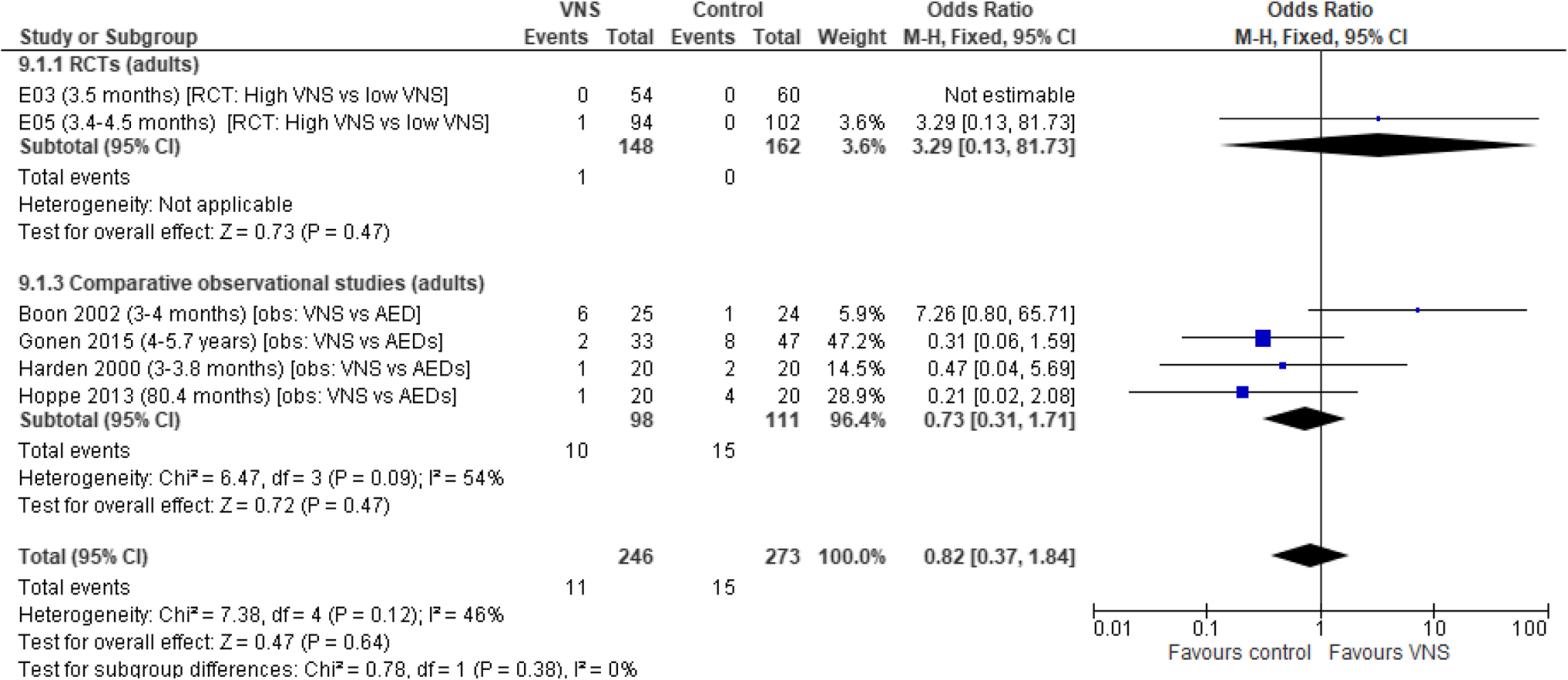
Seizure-free participants. Abbreviations: *ASM *antiepileptic drug,* CI *confidence interval,* RCT *randomised controlled trial,* SD *standard deviation,* VNS *Vagus Nerve Stimulation

A total of six studies (two RCTs and four comparative observational studies) were included in the analysis. There is no difference in the odds of freedom from seizures in adult participants undergoing VNS Therapy compared with low-stimulation VNS Therapy/ASM (OR: 0.82 [95% CI 0.37, 1.84]; *p* = 0.64). On a study level, results were inconsistent across RCTs and comparative observational studies. Moderate levels of heterogeneity were observed between studies and there were large levels of uncertainty across the trial estimates due to low event numbers (Fig. 4).

### Mean change from baseline in seizure frequency

**Fig. 5:**
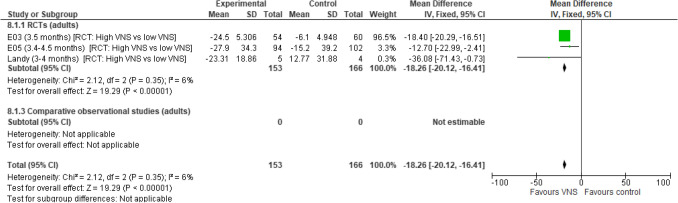
Change from baseline in seizures, percentage. Abbreviations: *ASM *antiepileptic drug, *CI *confidence interval,* RCT *randomised controlled trial,* SD *standard deviation,* VNS *Vagus Nerve Stimulation

Three RCT studies were included in the analysis. VNS Therapy was associated with a statistically significant decrease in the percentage change from baseline in seizures compared with low VNS Therapy (CFB:  – 18.26% [95% CI  – 20.12,  – 16.41]; *p* < 0.00001). Consistent results were observed across the three RCTs reporting on this outcome and low levels of heterogeneity were observed (Fig. 5).

### ASM load

**Fig. 6:**
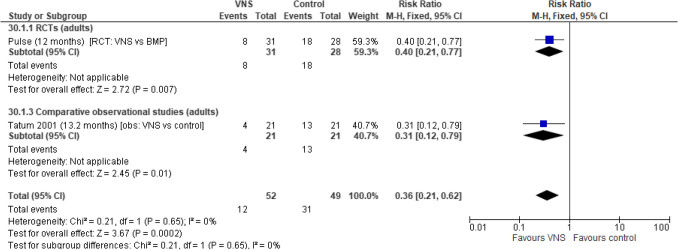
Number of participants with increased ASM load. Abbreviations: *ASM *anti-seizure medication, *CI *confidence interval,* RCT *randomised controlled trial, *VNS *Vagus Nerve Stimulation

**Fig. 7:**
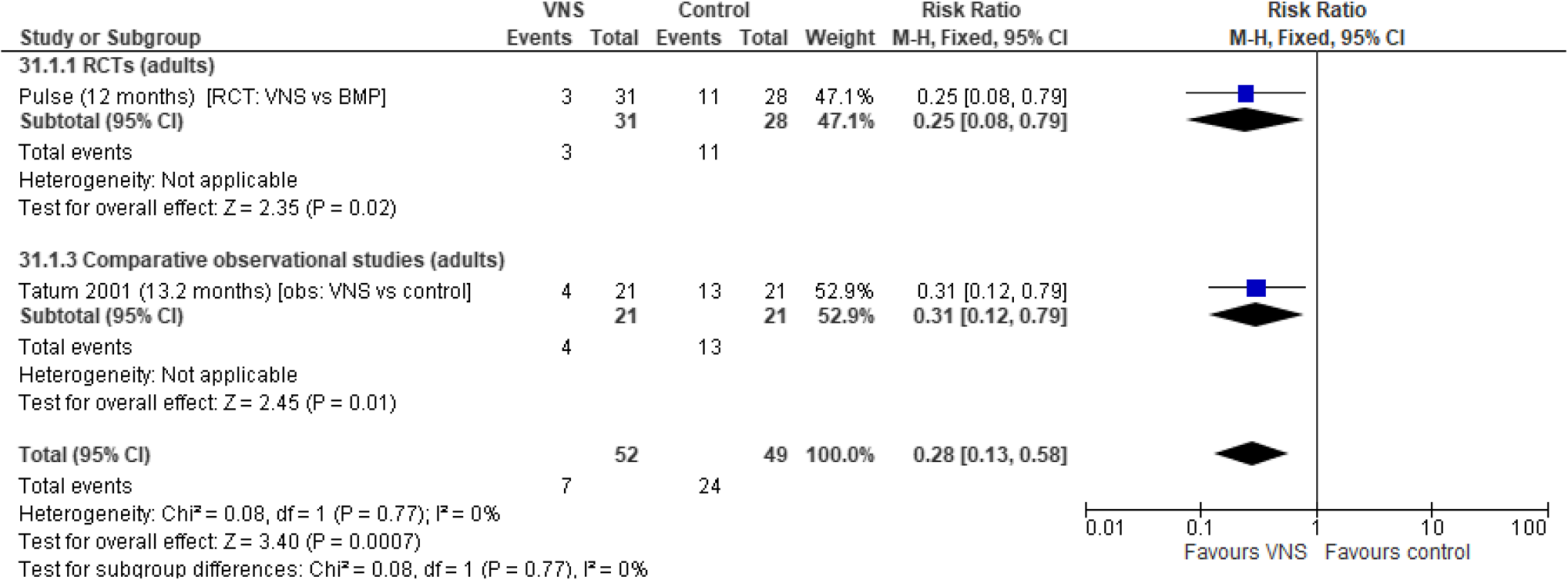
Number of participants with one or more new ASMs. Abbreviations: *ASM *anti-seizure medication,* CI *confidence interval,* RCT *randomised controlled trial,* VNS *Vagus Nerve Stimulation

The analysis for ASM load was based on two studies (one RCTs and one comparative observational studies). In the pooled analysis, participants undergoing VNS Therapy had a significant reduction in the risk of having an increased ASM load when compared with BMP or control (case-matched participants on ASMs) (risk ratio [RR]: 0.36 [95% CI 0.21, 0.62]; *p* = 0.0002). Similarly, pooled analysis indicated that participants undergoing VNS Therapy had a significant reduction in the risk of adding one or more new ASMs during treatment when compared with BMP or control (case-matched participants on ASMs) (RR: 0.28 [95% CI 0.13, 0.58]; *p* = 0.0007). Results from a single RCT and comparative observational study formed the pooled analysis for both outcomes; low levels of heterogeneity were observed between studies. Separately, both studies reported significant differences for both outcomes favouring VNS Therapy (see Figs. 6 and 7).

### VNS Therapy discontinuation

**Fig. 8:**
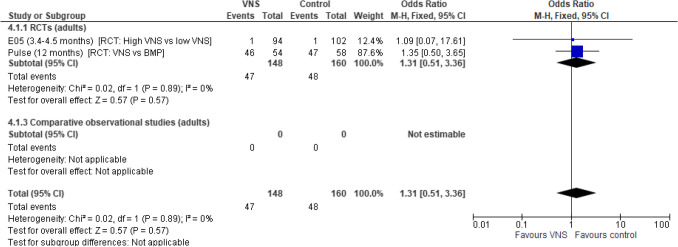
Treatment discontinuations. Abbreviations: *ASM, anti-seizure medication; BMP, best medical practice; CI, confidence interval; RCT, randomised controlled trial; VNS, Vagus Nerve Stimulation*

The discontinuation analysis included two RCT studies; no difference in the odds of discontinuing VNS Therapy treatment in adult participants undergoing VNS Therapy versus low-stimulation VNS Therapy/BMP was observed (OR: 1.31 [95% CI 0.51, 3.36]; *p* = 0.57). Consistent results were observed across the two RCTs reporting on this outcome. Low levels of heterogeneity were observed between studies and there were large levels of uncertainty across the trial estimates due to low event numbers (Fig. 8).

### SAEs

**Fig. 9:**
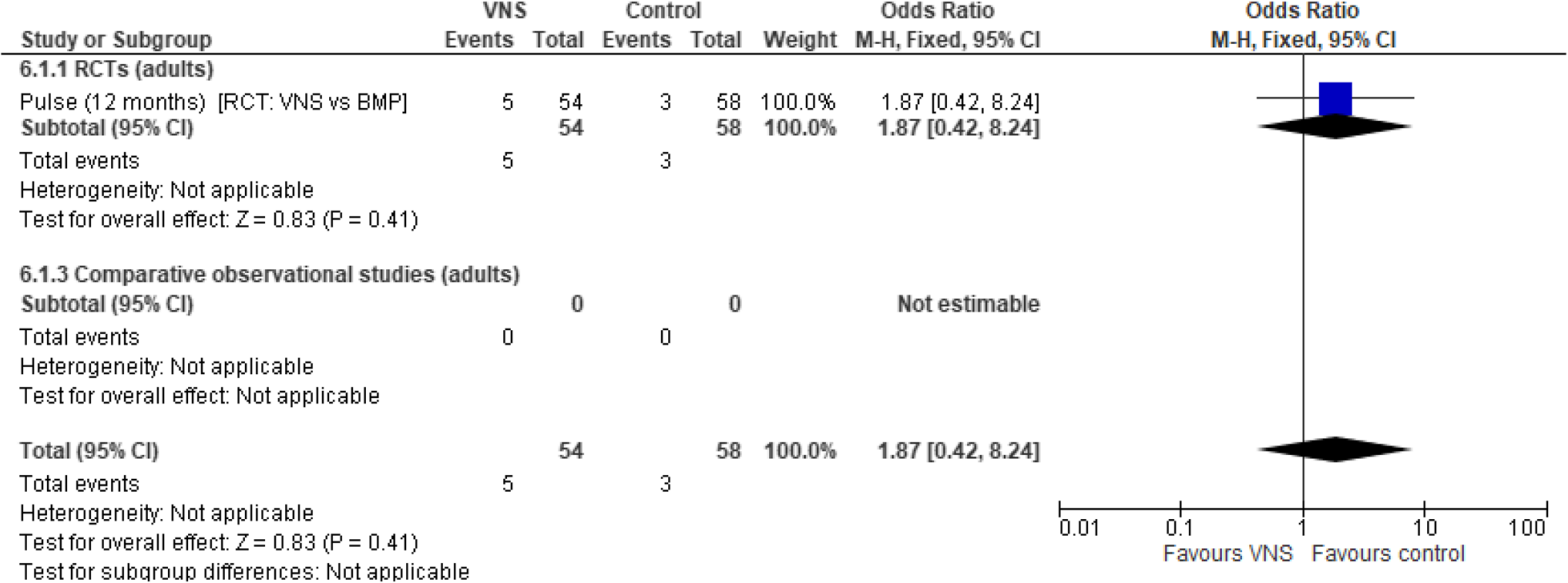
SAEs. Abbreviations: *ASM *anti-seizure medication,* BMP *best medical practice,* CI *confidence interval,* RCT *randomised controlled trial,* SAE *serious adverse event,* VNS *Vagus Nerve Stimulation

A single RCT study was included in the SAE analysis. No difference in the odds of an SAE in adult participants undergoing VNS Therapy compared with BMP was observed (OR: 1.87 [95% CI 0.42, 8.24]; *p* = 0.41) (Fig. 9).

## Discussion

This systematic review and meta-analysis demonstrated that in people with DRE, adjunctive high-stimulation VNS Therapy resulted in statistically significant reductions in seizure frequency without increasing the rate of SAEs or discontinuations when compared with adjunctive low-stimulation VNS Therapy/ASM/best medical practice. This evidence validates the consideration of VNS Therapy for people who respond poorly to ASMs, or those who are unsuitable for or unwilling to undergo any cranial procedure. Furthermore, the results of this study are in agreement with the current guideline recommendations for the use of VNS Therapy in adults [27, 70–72].

While VNS Therapy resulted in a statistically significant outcomes at the pooled level, some were not statistically significant at the trial level. For the ≥ 50% reduction in seizure frequency outcome, only a single trial was statistically significant at the trial level (E03). The other studies (E-05 and PuLsE) were not statistically significant likely due to the low number of participants involved and wide confidence intervals observed. For the ≥ 75% reduction in seizure frequency outcome, the pooled analysis (RCTs and comparative observational studies) and pooled RCT analysis both reported a statistically significant benefit of VNS Therapy. However, the pooled results for the comparative observational studies were not statistically significant, possibly due to study heterogeneity (specifically participant number, study length) and different magnitudes of treatment effects.

There is no difference in the odds of complete freedom from seizures for adult participants undergoing VNS Therapy versus low-stimulation VNS Therapy/ASM. This result reflects current evidence in the literature, with other studies reporting that people with DRE undergoing VNS Therapy have a low rate of seizure freedom, despite response and seizure freedom rates increasing over time [73]. It must be noted that no events for seizure freedom were observed in RCT studies included in this analysis, with seizure freedom events only recorded in the comparative observational studies, which have a longer follow-up. Seizure freedom, however, was observed in 15 of 273 individuals with DRE.

The beneficial impact of VNS Therapy on ASM load was limited to two studies (PuLsE RCT and Tatum 2001), indicating that participants are less likely to require new ASMs or have an increased ASM load compared with BMP or control (case-matched participants on ASMs). When viewed alongside other seizure control outcomes from this analysis, the evidence suggests that VNS Therapy may permit the reduction in concomitant ASMs without loss of seizure control. A lower drug burden is clinically important, because excessive drug load may be associated with decreased tolerability, and may consequently reduce the likelihood of seizure freedom [74]. Furthermore, certain ASMs are linked with a range of metabolic consequences that can adversely affect bone, lipid, and gonadal steroid metabolism. Consequently, reducing the drug burden may lower the risk of such complications [75]. Reductions in ASM load may also improve participant QoL, as a greater number of ASMs is a significant predictor of poor QoL [76]. In addition, studies have shown that seizure frequency in people with DRE was one of the most important factors contributing to patient QoL [77, 78]. Consequently, a reduction in seizures and their frequency may translate into QoL benefits. Of note, several studies which investigated use of VNS Therapy in individuals with DRE report improvements in seizure control and also observed improvements in QoL [35, 67, 79].

VNS therapy has comparable safety outcomes, specifically for SAEs and discontinuations, when compared with low-stimulation VNS Therapy/best medical practice. There was no difference in the odds of discontinuing treatment in adult participants undergoing VNS Therapy versus low-stimulation VNS Therapy/best medical practice, and there was no difference in the odds of an SAE in adult participants undergoing VNS Therapy versus best medical practice. When viewed alongside the seizure control outcomes from this analysis, the safety evidence suggests that VNS Therapy may facilitate better seizure control without increasing the rate of discontinuation or SAEs compared with participants undergoing VNS Therapy versus low-stimulation VNS Therapy/best medical practice. The discontinuation analysis was based on two RCTs of different duration; 12 months (PuLsE) and 3.4–4.5 months (E-05). Of note, there was only a single event in the VNS Therapy and comparator arm for E-05 compared with 46 and 47 events in the VNS Therapy and comparator arm for PuLsE. The main reasons for discontinuation in E-05 were Cheyne–Stokes respiration (*n* = 1), and a variety of unspecified symptoms (*n* = 1). For PuLsE, the majority of study discontinuations in either treatment group were due to premature termination of the study by the sponsor, and there were no discontinuations due to AEs.

It must be noted that of the studies identified for the meta-analysis, there was only one RCT (PuLsE; which had its outcomes restricted to 12 months for the meta-analysis) [35] and two comparative observational studies [63, 64] which reported long-term outcomes (≥ 2 years). Consequently, this makes it difficult to determine the long-term benefits associated with VNS Therapy. However, there are non-comparative, single-arm studies of VNS Therapy in people with DRE which provide an insight into the long-term treatment effects of VNS Therapy. A retrospective analysis of 436 participants (predominantly adults) with DRE treated with VNS Therapy reported that participants achieved a mean seizure reduction of 55.8% after a mean follow-up of 5 years; 40.5 and 63.75% of participants achieved ≥ 75% seizure control and ≥ 50% seizure control, respectively [80]. The mean reduction in seizures continued to improve with duration; of those participants with > 10 years of follow-up (*n* = 65), the mean decrease in seizure frequency at last follow-up was 76.3% [81]. In addition, results from a prospective, open-label study of long-term VNS Therapy use (2 years) in individuals with DRE (*n* = 40) reported no significant safety events associated with Therapy and 95% (38/40) of patients remained on VNS Therapy for the study duration (one patient died [SUDEP] and the other was lost to follow-up after 1 year of treatment) [82]. The long-term benefits of VNS Therapy are reported in a number of other single-arm studies [83–85]. These results highlight the long-term benefits of VNS Therapy for people with DRE, but long-term comparative studies are required to determine if the benefits observed were solely due to VNS Therapy, or a potentially synergistic combination of ASM regimens and VNS Therapy. There are single-arm studies of shorter duration which support the meta-analysis results for VNS Therapy and ASM load. DeGiorgio et al. 2000 reported that participants with refractory epilepsy (*n* = 195) who were treated with VNS Therapy had a reduction in the mean number of ASMs, from 2.3 to 2.1 at the end of 12 months [46]. In addition, another study reported that up to 40% of participants experienced a decrease in the total dose of ASMs after 12 months of VNS Therapy [86]. While positive, these observations need to be supported by long-term comparative studies.

As with all systematic reviews and meta-analyses, the results may need to be interpreted with caution due to certain limitations which include inconsistency across the trials for length of follow-up, greater treatment effects were often observed with observational comparative studies versus RCTs, and there were a very limited number of studies (≤ 2) for certain meta-analysis outcomes, specifically the discontinuation, SAE, and ASM load analyses. Of note, the number of studies identified for the meta-analysis was limited as the analysis focused on comparative observational studies and RCTs (which are the gold standard for generating estimates of relative treatment effects) which can be viewed as a strength of this analysis. Overall, there is limited high-quality evidence supporting the use of VNS Therapy in DRE. In addition, many trial-level estimates are associated with large levels of uncertainty (wide CIs) due to low participant and event numbers and in some instances single events are driving the direction of treatment effects. There was substantial variation in baseline seizure frequency reported by observational comparative studies (0.1–3.5 seizures per day). Seizure frequency in VNS Therapy participants and control participants were not comparable at baseline in the majority of reporting studies, with participants in the VNS Therapy arm having a greater baseline seizure frequency [63, 66, 67]. Another limitation of the analysis was the differences in VNS Therapy stimulation parameters across studies contributing to further heterogeneity amongst participant groups. In the early RCTs regulating stimulation parameters, the low-stimulation group was titrated to sensation and the high stimulation group to maximum tolerated stimulation. Subsequent studies have suggested this may not be necessary for optimal efficacy and may contribute to difficulties in tolerability. Finally, three of the VNS Therapy trials informing efficacy (E-03, E-05 and Landy 1993) did not compare VNS Therapy with ASM therapy only. These trials compared VNS Therapy at ‘high stimulation’ settings with a presumed sub-therapeutic ‘low-stimulation’ regimen; ASMs were given in both arms. Therapeutic VNS is driven by the generation of action potentials along the vagus nerve, which is a function of the strength-duration relationship [87]. It is reported in the literature that 1.5 mA at 130 µsec to 2.25 mA at 500 µsec is considered a therapeutic dose [20, 62, 69, 88]. Based on the reported data, the low-stimulation arms in each of these trials contain patients that could fall within this therapeutic range (see Table 3). Consequently, any residual benefit of ‘low-stimulation’ may have resulted in the overestimation of the efficacy of ASMs in the low-stimulation group.

This study has highlighted areas of focus for future research. There is a need for comparative studies assessing the long-term efficacy and safety of VNS Therapy as an adjunct to ASMs compared with relevant comparators. In addition, more research is required to reinforce the positive results observed for ASM load when VNS Therapy is used as an adjunct to ASMs.

## Conclusions

Although there is much literature devoted to VNS Therapy, there is a paucity of comparative data and this should be a focus for future research. This meta-analysis demonstrated the benefits of VNS Therapy in people with DRE, which included improvement in seizure frequency without an increase in the rate of SAEs or discontinuations. The evidence validates the consideration of VNS Therapy for people who are not responding to ASMs, or those who are unsuitable for or unwilling to undergo cranial procedures.

## Supplementary Information

Below is the link to the electronic supplementary material.

Supplementary file1 (DOCX 86 KB)
