# Supplementary material for: Efficacy and safety of VNS therapy or continued medication management for treatment of adults with drug-resistant epilepsy: systematic review and meta-analysis
Source: J Neurol. 2022 Jan 16;269(6):2874–91. doi: 10.1007/s00415-022-10967-6 (PMC9119900; doi:10.1007/s00415-022-10967-6)
Supplement: Supplementary file 1 — Supplementary file1 (DOCX 86 KB) [file 415_2022_10967_MOESM1_ESM.docx]

Efficacy and Safety of VNS Therapy or Continued Medication Management for Treatment of Adults with Drug-Resistant Epilepsy

**Journal of Neurology**

Sarah Batson, PhD, Rohit Shankar, FRCPsych, Joan Conry, MD, Jane Boggs, MD, Rodney Radtke, MD, Stephen Mitchell, PhD, Francesca Barion, PhD, Joanna Murphy, Vanessa Danielson, MSc

Corresponding author:

Sarah Batson, Mtech Access Limited, 30 Murdock Road, Bicester, Oxfordshire, OX26 4PP, UK

Email: [sarah.batson@mtechaccess.co.uk](mailto:sarah.batson@mtechaccess.co.uk)

**Supplementary materials**

***Search strategies***

Embase 1974 to 2020 August 24

| **#** | **Searches** | **Results** |
| --- | --- | --- |
| 1 | exp epilepsy/ | 233757 |
| 2 | (epilep$ or seizure$ or convuls$ or vagal or ilae).mp. | 409791 |
| 3 | tuberous sclerosis.mp. or exp tuberous sclerosis/ | 13036 |
| 4 | Lennox Gastaut syndrome/ or Lennox Gastaut*.mp. | 4195 |
| 5 | status epilepticus.mp. or epileptic state/ | 25893 |
| 6 | (dravet* adj2 syndrome).mp. | 1757 |
| 7 | exp severe myoclonic epilepsy in infancy/ | 1878 |
| 8 | 1 or 2 or 3 or 4 or 5 or 6 or 7 | 431053 |
| 9 | exp vagus nerve/ | 18636 |
| 10 | exp electrotherapy/ | 237832 |
| 11 | exp vagus nerve stimulation/ | 10549 |
| 12 | exp brain depth stimulation/ | 42351 |
| 13 | exp transcranial direct current stimulation/ | 7174 |
| 14 | exp transcranial magnetic stimulation/ | 24565 |
| 15 | exp implantable neurostimulator/ | 2972 |
| 16 | exp electrostimulation/ | 69247 |
| 17 | exp electrode implant/ | 2743 |
| 18 | (trigeminal nerve adj8 stimulat*).mp. | 1059 |
| 19 | (vagus nerve adj8 stimulat*).mp. | 12235 |
| 20 | (vagal nerve adj8 stimulat*).mp. | 2149 |
| 21 | vns.mp. | 3594 |
| 22 | neurostimulat*.mp. | 6379 |
| 23 | (cyberonics or livanova).mp. | 667 |
| 24 | NeuroCybernetic Prosthes*.mp. | 63 |
| 25 | ncp.mp. | 2008 |
| 26 | electric* stimulat*.mp. | 69095 |
| 27 | (electric* nerve adj5 stimulat*).mp. | 5271 |
| 28 | (cardiac based seizure*1 adj5 detect*).mp. | 18 |
| 29 | (Cbsd or dbs or rns or eTNS or TNS or TMS or rTMS or DCS or tDCS).mp. | 93423 |
| 30 | (autostim* or aspiresr*).mp. | 374 |
| 31 | ((deep brain or magnetic or transcranial or direct current or automatic or close loop or responsive) adj5 (stimulat* or brain pacemaker* or brain pace maker*)).mp. | 61833 |
| 32 | exp ketogenic diet/ | 5387 |
| 33 | (keto* adj3 (diet* or therap*)).mp. | 7864 |
| 34 | ((low* or poor or restrict*) adj carbohydrate*).mp. | 6063 |
| 35 | exp Atkins diet/ | 246 |
| 36 | (atkin* adj3 (diet* or therap*)).mp. | 589 |
| 37 | exp fenfluramine/ | 6433 |
| 38 | (fenfluramine or Pondimin* or Fintepla*).mp. | 6983 |
| 39 | exp cenobamate/ | 67 |
| 40 | (Cenobamate or Xcopri* or YKP3089 or YKP 3089).mp. | 81 |
| 41 | cannabidiol/ | 4880 |
| 42 | (Cannabidiol or Epidiolex*).mp. | 5678 |
| 43 | exp everolimus/ | 26905 |
| 44 | (Everolimus or everolimus or Afinitor* or Votubia*).mp. | 30126 |
| 45 | exp brivaracetam/ | 703 |
| 46 | (brivaracetam or Briviact*).mp. | 726 |
| 47 | lobectomy/ or lobectom*.mp. | 41503 |
| 48 | (cort* adj2 excis*).mp. | 248 |
| 49 | (hemispher* adj2 disconnect*).mp. | 236 |
| 50 | hemispherectomy.mp. or exp hemispherectomy/ | 2058 |
| 51 | (surg* adj8 epilep*).mp. | 17353 |
| 52 | random*.ti,ab. | 1565492 |
| 53 | factorial*.ti,ab. | 38721 |
| 54 | (crossover* or cross over*).ti,ab. | 107948 |
| 55 | ((doubl* or singl*) adj blind*).ti,ab. | 235331 |
| 56 | (assign* or allocat* or volunteer* or placebo*).ti,ab. | 1050695 |
| 57 | crossover procedure/ | 64163 |
| 58 | double blind procedure/ | 175318 |
| 59 | single blind procedure/ | 39942 |
| 60 | randomized controlled trial/ | 616932 |
| 61 | exp controlled clinical trial/ | 804264 |
| 62 | or/52-61 | 2506671 |
| 63 | register/ | 113842 |
| 64 | registr*.ti,ab. | 341206 |
| 65 | clinical study/ | 155681 |
| 66 | prospective study/ | 621483 |
| 67 | retrospective study/ | 952869 |
| 68 | (prospective* or retrospective*).ti,ab. | 2222818 |
| 69 | 66 or 67 or 68 | 2506581 |
| 70 | 65 and 69 | 22787 |
| 71 | 62 or 63 or 64 or 70 | 2812526 |
| 72 | 9 or 10 or 11 or 12 or 13 or 14 or 15 or 16 or 17 or 18 or 19 or 20 or 21 or 22 or 23 or 24 or 25 or 26 or 27 or 28 or 29 or 30 or 31 or 32 or 33 or 34 or 35 or 36 or 37 or 38 or 39 or 40 or 41 or 42 or 43 or 44 or 45 or 46 or 47 or 48 or 49 or 50 or 51 | 517467 |
| 73 | 8 and 71 and 72 | 5523 |

Ovid MEDLINE(R) and Epub Ahead of Print, In-Process & Other Non-Indexed Citations, Daily and Versions(R) 1946 to August 24, 2020

| **#** | **Searches** | **Results** |
| --- | --- | --- |
| 1 | exp Epilepsy/ | 111626 |
| 2 | (epilep$ or seizure$ or convuls$ or vagal or ilae).mp. | 266873 |
| 3 | tuberous sclerosis.mp. or exp Tuberous Sclerosis/ | 9770 |
| 4 | exp Lennox Gastaut Syndrome/ or Lennox Gastaut*.mp. | 1484 |
| 5 | status epilepticus.mp. or exp Status Epilepticus/ | 13803 |
| 6 | dravet syndrome.mp. or exp Epilepsies, Myoclonic/ | 5164 |
| 7 | 1 or 2 or 3 or 4 or 5 or 6 | 275984 |
| 8 | exp Vagus Nerve/ | 26605 |
| 9 | exp Electric Stimulation Therapy/ | 80175 |
| 10 | exp Vagus Nerve Stimulation/ | 1565 |
| 11 | exp Deep Brain Stimulation/ | 8716 |
| 12 | exp Transcranial Direct Current Stimulation/ | 2672 |
| 13 | exp Transcranial Magnetic Stimulation/ | 11490 |
| 14 | exp Implantable Neurostimulators/ | 11318 |
| 15 | exp Electric Stimulation/ | 126731 |
| 16 | exp Electrodes, Implanted/ | 46198 |
| 17 | (trigeminal nerve adj8 stimulat*).mp. | 675 |
| 18 | (vagus nerve adj8 stimulat*).mp. | 4327 |
| 19 | (vagal nerve adj8 stimulat*).mp. | 1342 |
| 20 | vns.mp. | 2049 |
| 21 | neurostimulat*.mp. | 3765 |
| 22 | (cyberonics or livanova).mp. | 132 |
| 23 | NeuroCybernetic Prosthes*.mp. | 27 |
| 24 | ncp.mp. | 1491 |
| 25 | electric* stimulat*.mp. | 153916 |
| 26 | (electric* nerve adj5 stimulat*).mp. | 6260 |
| 27 | (cardiac based seizure*1 adj5 detect*).mp. | 4 |
| 28 | (Cbsd or dbs or rns or eTNS or TNS or TMS or rTMS or DCS or tDCS).mp. | 62798 |
| 29 | (autostim* or aspiresr*).mp. | 320 |
| 30 | ((deep brain or magnetic or transcranial or direct current or automatic or close loop or responsive) adj5 (stimulat* or brain pacemaker* or brain pace maker*)).mp. | 40898 |
| 31 | exp Diet, Ketogenic/ | 1327 |
| 32 | (keto* adj3 (diet* or therap*)).mp. | 4415 |
| 33 | ((low* or poor or restrict*) adj carbohydrate*).mp. | 3347 |
| 34 | exp Diet, High-Protein Low-Carbohydrate/ | 42 |
| 35 | (atkin* adj3 (diet* or therap*)).mp. | 327 |
| 36 | exp Fenfluramine/ | 3060 |
| 37 | (fenfluramine or Pondimin* or Fintepla*).mp. | 3677 |
| 38 | (Cenobamate or Xcopri* or YKP3089 or YKP 3089).mp. | 27 |
| 39 | exp Cannabidiol/ | 1659 |
| 40 | (Cannabidiol or Epidiolex*).mp. | 3210 |
| 41 | exp Everolimus/ | 4747 |
| 42 | (Everolimus or everolimus or Afinitor* or Votubia*).mp. | 7465 |
| 43 | (brivaracetam or Briviact*).mp. | 280 |
| 44 | exp Anterior Temporal Lobectomy/ | 813 |
| 45 | lobectom*.mp. | 20143 |
| 46 | (cort* adj2 excis*).mp. | 210 |
| 47 | (hemispher* adj2 disconnect*).mp. | 171 |
| 48 | hemispherectomy.mp. or exp Hemispherectomy/ | 1440 |
| 49 | (surg* adj8 epilep*).mp. | 10176 |
| 50 | 8 or 9 or 10 or 11 or 12 or 13 or 14 or 15 or 16 or 17 or 18 or 19 or 20 or 21 or 22 or 23 or 24 or 25 or 26 or 27 or 28 or 29 or 30 or 31 or 32 or 33 or 34 or 35 or 36 or 37 or 38 or 39 or 40 or 41 or 42 or 43 or 44 or 45 or 46 or 47 or 48 or 49 | 392265 |
| 51 | randomized controlled trial.pt. | 511654 |
| 52 | controlled clinical trial.pt. | 93813 |
| 53 | randomi#ed.ab. | 586555 |
| 54 | placebo.ab. | 210465 |
| 55 | randomly.ab. | 339616 |
| 56 | clinical trials as topic.sh. | 192618 |
| 57 | trial.ti. | 223917 |
| 58 | 51 or 52 or 53 or 54 or 55 or 56 or 57 | 1346171 |
| 59 | Registries/ | 89914 |
| 60 | registr*.ti,ab. | 246715 |
| 61 | Clinical Study/ | 3606 |
| 62 | Prospective Studies/ | 546192 |
| 63 | Retrospective Studies/ | 834105 |
| 64 | (prospective* or retrospective*).ti,ab. | 1393427 |
| 65 | 62 or 63 or 64 | 1863449 |
| 66 | 61 and 65 | 1959 |
| 67 | 58 or 59 or 60 or 66 | 1562140 |
| 68 | 7 and 50 and 67 | 2078 |

EBM Reviews - Cochrane Database of Systematic Reviews 2005 to August 20, 2020, EBM Reviews - ACP Journal Club 1991 to July 2020, EBM Reviews - Database of Abstracts of Reviews of Effects 1st Quarter 2016, EBM Reviews - Cochrane Clinical Answers August 2020, EBM Reviews - Cochrane Central Register of Controlled Trials July 2020, EBM Reviews - Cochrane Methodology Register 3rd Quarter 2012, EBM Reviews - Health Technology Assessment 4th Quarter 2016, EBM Reviews - NHS Economic Evaluation Database 1st Quarter 2016

| **#** | **Searches** | **Results** |
| --- | --- | --- |
| 1 | exp Epilepsy/ | 3100 |
| 2 | (epilep$ or seizure$ or convuls$ or vagal or ilae).mp. | 17403 |
| 3 | tuberous sclerosis.mp. or exp Tuberous Sclerosis/ | 265 |
| 4 | exp Lennox Gastaut Syndrome/ or Lennox Gastaut*.mp. | 283 |
| 5 | status epilepticus.mp. or exp Status Epilepticus/ | 498 |
| 6 | dravet syndrome.mp. or exp Epilepsies, Myoclonic/ | 148 |
| 7 | 1 or 2 or 3 or 4 or 5 or 6 | 17584 |
| 8 | exp Vagus Nerve/ | 402 |
| 9 | exp Electric Stimulation Therapy/ | 6473 |
| 10 | exp Vagus Nerve Stimulation/ | 86 |
| 11 | exp Deep Brain Stimulation/ | 317 |
| 12 | exp Transcranial Direct Current Stimulation/ | 0 |
| 13 | exp Transcranial Magnetic Stimulation/ | 1402 |
| 14 | exp Implantable Neurostimulators/ | 191 |
| 15 | exp Electric Stimulation/ | 1976 |
| 16 | exp Electrodes, Implanted/ | 1685 |
| 17 | (trigeminal nerve adj8 stimulat*).mp. | 91 |
| 18 | (vagus nerve adj8 stimulat*).mp. | 812 |
| 19 | (vagal nerve adj8 stimulat*).mp. | 186 |
| 20 | vns.mp. | 398 |
| 21 | neurostimulat*.mp. | 907 |
| 22 | (cyberonics or livanova).mp. | 24 |
| 23 | NeuroCybernetic Prosthes*.mp. | 8 |
| 24 | ncp.mp. | 78 |
| 25 | electric* stimulat*.mp. | 9202 |
| 26 | (electric* nerve adj5 stimulat*).mp. | 3076 |
| 27 | (cardiac based seizure*1 adj5 detect*).mp. | 2 |
| 28 | (Cbsd or dbs or rns or eTNS or TNS or TMS or rTMS or DCS or tDCS).mp. | 10529 |
| 29 | (autostim* or aspiresr*).mp. | 3 |
| 30 | ((deep brain or magnetic or transcranial or direct current or automatic or close loop or responsive) adj5 (stimulat* or brain pacemaker* or brain pace maker*)).mp. | 11070 |
| 31 | exp Diet, Ketogenic/ | 2 |
| 32 | (keto* adj3 (diet* or therap*)).mp. | 1645 |
| 33 | ((low* or poor or restrict*) adj carbohydrate*).mp. | 1440 |
| 34 | exp Diet, High-Protein Low-Carbohydrate/ | 0 |
| 35 | (atkin* adj3 (diet* or therap*)).mp. | 127 |
| 36 | exp Fenfluramine/ | 378 |
| 37 | (fenfluramine or Pondimin* or Fintepla*).mp. | 555 |
| 38 | (Cenobamate or Xcopri* or YKP3089 or YKP 3089).mp. | 29 |
| 39 | exp Cannabidiol/ | 124 |
| 40 | (Cannabidiol or Epidiolex*).mp. | 640 |
| 41 | exp Everolimus/ | 0 |
| 42 | (Everolimus or everolimus or Afinitor* or Votubia*).mp. | 4459 |
| 43 | (brivaracetam or Briviact*).mp. | 152 |
| 44 | exp Anterior Temporal Lobectomy/ | 23 |
| 45 | lobectom*.mp. | 1642 |
| 46 | (cort* adj2 excis*).mp. | 12 |
| 47 | (hemispher* adj2 disconnect*).mp. | 4 |
| 48 | hemispherectomy.mp. or exp Hemispherectomy/ | 8 |
| 49 | (surg* adj8 epilep*).mp. | 533 |
| 50 | 8 or 9 or 10 or 11 or 12 or 13 or 14 or 15 or 16 or 17 or 18 or 19 or 20 or 21 or 22 or 23 or 24 or 25 or 26 or 27 or 28 or 29 or 30 or 31 or 32 or 33 or 34 or 35 or 36 or 37 or 38 or 39 or 40 or 41 or 42 or 43 or 44 or 45 or 46 or 47 or 48 or 49 | 40224 |
| 51 | 7 and 50 | 2576 |

Supplementary Materials Table 1: List of VNS publications identified in the SLR

| Study name | Primary study | Included in meta-analysis? | Linked publications | Included meta-analysis? |
| --- | --- | --- | --- | --- |
| **VNS RCTs (unique studies, n=7 [publications, n=24])** | | | | |
| Klinkenberg 2012 | Klinkenberg S et al. Vagus nerve stimulation in children with intractable epilepsy: a randomized controlled trial. Dev Med Child Neurol. 2012 Sep;54(9):855-61. | No | Aalbers MW et al. The effects of vagus nerve stimulation on pro- and anti-inflammatory cytokines in children with refractory epilepsy: an exploratory study.  Neuroimmunomodulation. 2012;19(6):352-8. | No |
|  |  |  | Klinkenberg S et al. Behavioural and cognitive effects during vagus nerve stimulation in children with intractable epilepsy - a randomized controlled trial. Eur J Paediatr Neurol. 2013; Jan;17(1):82-90. | No |
|  |  |  | The effects of vagus nerve stimulation on tryptophan metabolites in children with intractable epilepsy. Epilepsy Behav. 2014 Aug;37:133-8. | No |
| *E03* | Salinsky MC. A randomized controlled trial of chronic vagus nerve stimulation for treatment of medically intractable seizures. Neurology. 1995;45(2):224-30. | Yes | Ben-Menachem E et al. Vagus nerve stimulation for treatment of partial seizures: 1. A controlled study of effect on seizures. First International Vagus Nerve Stimulation Study Group. Epilepsia. 1994;35(3):616-26. | No |
|  |  |  | Ramsay RE et al. Vagus nerve stimulation for treatment of partial seizures: 2. Safety, side effects, and tolerability. First International Vagus Nerve Stimulation Study Group. Epilepsia. 1994;35(3):627-36. | No |
|  |  |  | Holder LK et al. Treatment of refractory partial seizures: preliminary results of a controlled study. Pacing Clin Electrophysiol. 1992;15(10 Pt 2):1557-71. | No |
|  |  |  | Morris IGL. A retrospective analysis of the effects of magnet-activated stimulation in conjunction with vagus nerve stimulation therapy. Epilepsy and Behavior. 2003;4(6):740-5. | No |
|  |  |  | Michael JE et al. Vagus nerve stimulation for intractable seizures: one year follow-up. J Neurosci Nurs. 1993;25(6):362-6. | No |
|  |  |  | George R et al. Vagus nerve stimulation for treatment of partial seizures: 3. Long-term follow-up on first 67 patients exiting a controlled study. First International Vagus Nerve Stimulation Study Group. Epilepsia. 1994;35(3):637-43. | No |
|  |  |  | Holder LK. Long-term follow-up of 37 patients with refractory partial seizures treated with vagus nerve-stimulation. Journal of Epilepsy. 1992;6(4):206-14. | No |
|  |  |  | Salinsky MC et al. Vagus nerve stimulation for the treatment of medically intractable seizures. Results of a 1-year open-extension trial. Vagus Nerve Stimulation Study Group. Arch Neurol. 1996;53(11):1176-80. | No |
| E05 | Handforth A et al. Vagus nerve stimulation therapy for partial-onset seizures: A randomized active-control trial. Neurology. 1998;51(1):48-55. | Yes | Amar AP et al. An institutional experience with cervical vagus nerve trunk stimulation for medically refractory epilepsy: Rationale, technique, and outcome. Neurosurgery. 1998;43(6):1265-80. | No |
|  |  |  | Dodrill CB & Morris GL. Effects of Vagal Nerve Stimulation on Cognition and Quality of Life in Epilepsy. Epilepsy Behav. 2001;2(1):46-53. | No |
|  |  |  | Amar AP et al. Long-term multicenter experience with vagus nerve stimulation for intractable partial seizures: Results of the XE5 trial. Stereotactic and Functional Neurosurgery. 1999;73(1-4):104-8. | No |
|  |  |  | DeGiorgio CM et al. Prospective long-term study of vagus nerve stimulation for the treatment of refractory seizures. Epilepsia. 2000;41(9):1195-200. | No |
|  |  |  | DeGiorgio CM, Thompson J, Lewis P, Arrambide S, Naritoku D, Handforth A, et al. Vagus nerve stimulation: analysis of device parameters in 154 patients during the long-term XE5 study. Epilepsia. 2001;42(8):1017-20. | No |
| E06 | Bunker M. An Open Randomized Trial to Assess the Efficacy and Safety of Adjunctive Vagus Nerve Stimulation versus Adjunctive Anti-Epileptic Drug (AED) Treatment in Children with Refractory Seizures. 2012. | No | NA | NA |
| PuLse | Ryvlin P et al. The long-term effect of vagus nerve stimulation on quality of life in patients with pharmacoresistant focal epilepsy: the PuLsE (Open Prospective Randomized Long-term Effectiveness) trial. Epilepsia. 2014;55(6):893-900. | Yes | NA | NA |
| DeGiorgio 2005 | DeGiorgio C et al. Vagus nerve stimulation for epilepsy: randomized comparison of three stimulation paradigms. Neurology. 2005;65(2):317-9. | No | Bunch S et al. Vagus nerve stimulation for epilepsy: is output current correlated with acute response? Acta Neurol Scand. 2007;116(4):217-20. | No |
| Landy 1994 | Landy HJ et al. Vagus nerve stimulation for complex partial seizures: surgical technique, safety, and efficacy. J Neurosurg. 1993;78(1):26-31. | Yes | NA | NA |
| **VNS comparative observational studies (unique studies, n=23 [linked publications, n=24])** | | | | |
| Ben-Menachem 1995 | Ben-Menachem E et al. Effects of vagus nerve stimulation on amino acids and other metabolites in the CSF of patients with partial seizures. Epilepsy Res. 1995;20(3):221-7. | No | NA | NA |
| Bhraguvansh 2020 | Bhraguvansh A et al. VNS and RNS patients are driving: The impact of seizure freedom and reduction American Epilepsy Society. 2020;Abst. 154. | No | NA | NA |
| Boon 2002 | Boon P et al. Direct medical costs of refractory epilepsy incurred by three different treatment modalities: a prospective assessment. Epilepsia. 2002;43(1):96-102. | Yes | Boon P et al. Epilepsy surgery in Belgium, the experience in Gent. Acta Neurol Belg. 1999;99(4):256-65. | No |
| Drees 2018 | Drees C et al. Responsive neurostimulation (RNS) use in patients with and without prior vagus nerve stimulator (VNS) implantation. American Epilepsy Society. 2018;Abst. 1.165. | No | NA | NA |
| Ellens 2018 | Ellens NR et al. A Comparison of Vagal Nerve Stimulation and Responsive Neurostimulation for the Treatment of Medically Refractory Complex Partial Epilepsy. Stereotact Funct Neurosurg. 2018;96(4):259-63. | No | NA | NA |
| Gonen 2015 | Gonen OM. The prognosis of refractory epilepsy patients rejected from epilepsy surgery. Acta Neurol Scand. 2015;131(1):58-62 | Yes | NA | NA |
| Harden 2000 | Harden CL et al. A Pilot Study of Mood in Epilepsy Patients Treated with Vagus Nerve Stimulation. Epilepsy Behav. 2000;1(2):93-9. | No | NA | NA |
| Hoppe 2013 | Hoppe C et al. Comprehensive long-term outcome of best drug treatment with or without add-on vagus nerve stimulation for epilepsy: a retrospective matched pairs case-control study. Seizure. 2013;22(2):109-15. | Yes | NA | NA |
| Jamy 2019 | Jamy R et al. Practice trends and the outcome of neuromodulation therapies in epilepsy: A single-center study. Epilepsia Open. 2019;4(3):493-7. | No | NA | NA |
| Kim 2018 | Kim K et al. Retrospective comparison of seizure outcomes in patients with focal epilepsy treated with responsive neurostimulation or vagus nerve stimulation American Epilepsy Society. 2018;Abst. 3.199. | No | NA | NA |
| Kuba 2013 | Kuba R et al. Comparing the effects of cortical resection and vagus nerve stimulation in patients with nonlesional extratemporal epilepsy. Epilepsy Behav. 2013;28(3):474-80. | No | NA | NA |
| Lam 2020 | Lam S et al. Hospitalization costs and mortality in pediatric patients with drug-resistant epilepsy: Vagus nerve stimulation therapy versus medical management. American Epilepsy Society. 2020;Abst. 1012. | No | NA | NA |
| Lim 2018 | Lim Z et al. Vagus nerve stimulation for the treatment of refractory epilepsy in the CDKL5 Deficiency Disorder. Epilepsy Res. 2018;146:36-40. | No | NA | NA |
| Marrosu 2003 | Marrosu F et al. Correlation between GABA(A) receptor density and vagus nerve stimulation in individuals with drug-resistant partial epilepsy. Epilepsy Res. 2003;55(1-2):59-70. | Yes | NA | NA |
| Morrison-Levy 2018 | Morrison-Levy N et al. Children with autism spectrum disorders and drug-resistant epilepsy can benefit from epilepsy surgery. Epilepsy Behav. 2018;85:200-4. | No | NA | NA |
| McGlone 2008 | McGlone J et al. Quality of life and memory after vagus nerve stimulator implantation for epilepsy. Can J Neurol Sci. 2008;35(3):287-96. | No | NA | NA |
| Nei 2006 | Nei M et al. Refractory generalized seizures: response to corpus callosotomy and vagal nerve stimulation. Epilepsia. 2006;47(1):115-22. | No | NA | NA |
| Sherman 2008 | Sherman EM et al. Quality of life and seizure outcome after vagus nerve stimulation in children with intractable epilepsy. J Child Neurol. 2008;23(9):991-8. | No | NA | NA |
| Tatum 2001 | Tatum WO, Johnson KD, Goff S, Ferreira JA, Benbadis SR, Vale FL. Vagus nerve stimulation and drug reduction. Neurology. 2001 Feb 27;56(4):561-3. | Yes | NA | NA |
| Terra 2014 | Terra VC et al. Vagus nerve stimulation in pediatric patients: Is it really worthwhile? Epilepsy Behav. 2014;31:329-33. | No | NA | NA |
| Wang 2020 | Wang AJ et al. Vagus Nerve Stimulation versus Responsive Neurostimulator System in Patients with Temporal Lobe Epilepsy. Stereotact Funct Neurosurg. 2020;98(1):21-9. | No | NA | NA |
| CONTAIN | Wheless JW et al. Clobazam is efficacious for patients across the spectrum of disease severity of Lennox-Gastaut syndrome: post hoc analyses of clinical trial results by baseline seizure-frequency quartiles and VNS experience. Epilepsy Behav. 2014;41:47-52. | No | NA | NA |
| You 2008 | You SJ et al. Comparison of corpus callosotomy and vagus nerve stimulation in children with Lennox-Gastaut syndrome. Brain Dev. 2008;30(3):195-9. | No | NA | NA |
